# Efficient FPGA Implementation of Convolutional Neural Networks and Long Short-Term Memory for Radar Emitter Signal Recognition

**DOI:** 10.3390/s24030889

**Published:** 2024-01-30

**Authors:** Bin Wu, Xinyu Wu, Peng Li, Youbing Gao, Jiangbo Si, Naofal Al-Dhahir

**Affiliations:** 1 School of Electronic Engineering, Xidian University, Xi’an 710071, China; bwu@xidian.edu.cn (B.W.); penglixd@xidian.edu.cn (P.L.); 2 Science and Technology on Electronic Information Control Laboratory, Chengdu 610036, China; gaoyb@cetc.com; 3 Integrated Service Networks Laboratory, Xidian University, Xi’an 710071, China; jbsi@xidian.edu.cn; 4 Department of Electrical and Computer Engineering, The University of Texas at Dallas, Richardson, TX 75080, USA; aldhahir@utdallas.edu

**Keywords:** convolutional neural network (CNN), field programmable gate array (FPGA), hardware accelerators, long short-term memory (LSTM), radar emitter signal recognition

## Abstract

In recent years, radar emitter signal recognition has enjoyed a wide range of applications in electronic support measure systems and communication security. More and more deep learning algorithms have been used to improve the recognition accuracy of radar emitter signals. However, complex deep learning algorithms and data preprocessing operations have a huge demand for computing power, which cannot meet the requirements of low power consumption and high real-time processing scenarios. Therefore, many research works have remained in the experimental stage and cannot be actually implemented. To tackle this problem, this paper proposes a resource reuse computing acceleration platform based on field programmable gate arrays (FPGA), and implements a one-dimensional (1D) convolutional neural network (CNN) and long short-term memory (LSTM) neural network (NN) model for radar emitter signal recognition, directly targeting the intermediate frequency (IF) data of radar emitter signal for classification and recognition. The implementation of the 1D-CNN-LSTM neural network on FPGA is realized by multiplexing the same systolic array to accomplish the parallel acceleration of 1D convolution and matrix vector multiplication operations. We implemented our network on Xilinx XCKU040 to evaluate the effectiveness of our proposed solution. Our experiments show that the system can achieve 7.34 giga operations per second (GOPS) data throughput with only 5.022 W power consumption when the radar emitter signal recognition rate is 96.53%, which greatly improves the energy efficiency ratio and real-time performance of the radar emitter recognition system.

## 1. Introduction

Most of the traditional radar emitter signal (RES) identification methods are based on the RES parameters, such as carrier frequency, signal pulse width, signal amplitude, direction of arrival (DOA), and signal time of arrival (TOA). However, with the RES system and the modern electromagnetic environment becoming more complicated, the traditional identification method based on parameters cannot meet the requirements of RES identification requirements. In addition, whether in the field of electronic support measure systems or communications, RESs always need to be intentionally or unintentionally modulated before being radiated into space [1]. In recent years, according to this RES characteristic, there has been significant research on modulation type recognition, behavior recognition, and even specific RES identification using the methods of artificial intelligence and machine learning [2]. Compared with the traditional methods, recognition algorithms based on deep learning are widely used because of their powerful feature extraction ability, which greatly improves the RES recognition rate.

However, many recognition algorithms based on deep learning require complex RES preprocessing to complete the recognition task. For example, the RES is transformed into the frequency domain, time–frequency domain, or other transform domains, and then these features or images are classified and recognized after obtaining the transform domain features. In [3], the Hilbert transform and bispectrum of the RES are combined to form a signal image, which is fed into a convolutional neural network (CNN) to realize individual RES recognition. There are also other approaches to directly classify and identify RES intermediate frequency (IF) data using deep learning networks with complex structures, such as residual networks, attention mechanisms or hybrid neural network (NN) [4,5,6,7]. In [8], a combination of NN with an inception mechanism and long short-term memory (LSTM) neural networks was used to achieve individual identification of five universal software radio peripheral (USRP) communication emitter signal devices. Although such methods have high RES identification accuracy, the complex preprocessing process and algorithm model will inevitably lead to an increase in computing complexity and system latency, especially for power-limited computing devices, such as spaceborne devices or portable terminals, where most of the complex deep learning algorithms cannot be implemented. Therefore, in the design process of RES identification algorithms, more attention should be paid to the balance between signal recognition rate and algorithm complexity, and the implementation ability of an RES identification algorithm in engineering applications should not be ignored.

The rapid development of deep learning cannot be achieved without its powerful computing power, which is usually accelerated by parallel processors such as a graphics processing unit (GPU). Although a GPU can provide powerful computational support for deep learning algorithms, the huge power consumption of GPUs limits their application in low-power scenarios. An application-specific integrated circuit (ASIC) [9] can provide high-performance and low-power computational support for deep learning algorithms, but its high cost and long-term development process have been major deterrents. Hence, programmable logic gate arrays (FPGAs) have received increasing attention due to their reprogrammable, low power consumption, and abundant computational resources, and many FPGA-based computational acceleration schemes for deep learning algorithms have been proposed in recent years. A method to automatically deploy CNNs on on-board FPGAs was proposed in [10], which achieved 23.06 GOPS and 22.17 GOPS throughput rates for the simplified VGG16 network and YOLOv2 network deployed on a Xilinx AC701. In [11], the FPGA implementation of CNNs for radar signal processing was carefully optimized for better performance and energy efficiency. The authors of [12] achieved substantial improvements in computational speed and energy efficiency ratio of the LSTM network acceleration engine implemented on an FPGA compared to CPU and GPU using fixed-point parameters, systolic arrays, and nonlinear function lookup tables. In [13], an architecture for CNN implementation in FPGAs using the Winograd algorithm was proposed to reduce the complexity of convolutional operations and accelerate the computational process. In [14], a CNN acceleration was implemented specifically using deep separable convolution.

However, most of the current research on FPGA-based deep learning computational acceleration only focuses on the computational acceleration of one of the NN models, CNNs or RNNs [15], and cannot support the computation of C-RNN models combining a CNN and an RNN, and most of the related research focuses on the processing of two-dimensional image data.

**Figure 1:**
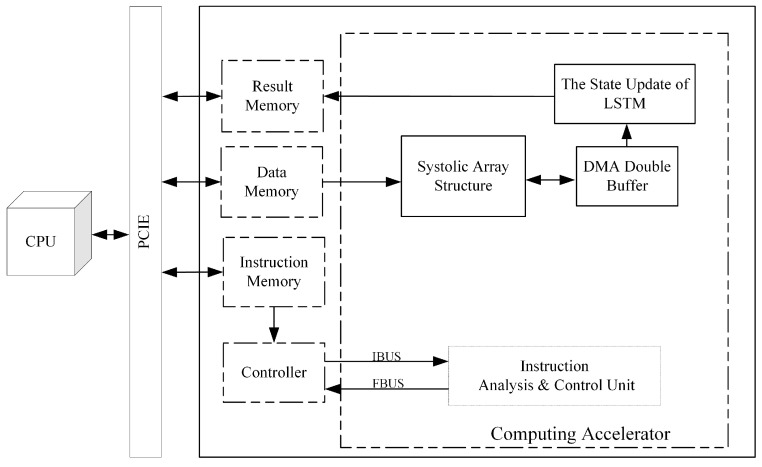
Resource multiplexing computational accelerator architecture.

Based on the above discussion, we constructed a one-dimensional (1D) CNN-LSTM model for RES classification and identification, considering the characteristics that the actual signal duration varies randomly and the information among sampling points is correlated in the time dimension. We exploited the different advantages of CNNs for reducing frequency variation, LSTM for temporal modeling, and deep NNs for mapping features to a more separable space [16], and designed an FPGA-based resource multiplexing computing acceleration platform as shown in Figure 1. The deployment of the CNN-LSTM algorithm model on FPGAs was realized, which enables the deep learning-based RES identification algorithm to be truly implemented in low-power and stringent real-time scenarios. The main contributions of this paper are summarized as follows:

1. We constructed a 1D-CNN-LSTM model for RES recognition, which can directly process IF data and guarantee high recognition accuracy with a simple structure that is more convenient for FPGA hardware implementation. Compared with a single CNN, the network model has no uniform requirement on the length of the input signal and is more suitable for processing pulse RESs with randomly varying length.
2. We designed an FPGA-based resource multiplexing computational acceleration platform for the 1D-CNN-LSTM model constructed in this paper, which achieves parallel acceleration of both 1D convolution and matrix multiplication operations by multiplexing the same systolic array, reducing the processing delay while greatly improving the utilization of FPGA computational resources.
3. For the different operation characteristics of CNNs and LSTM, a special instruction set of the FPGA acceleration platform was developed, which can realize rapid redeployment by adjusting instructions during the change of NN model structure or algorithm iteration.
4. On the Xilinx XKU040 FPGA development board, we have implemented a 1D-CNN-LSTM RES recognition system. The experimental results show that the system achieves a data throughput rate of up to 7.34 GOPS with a power consumption of only 5.022 W with a recognition rate of 97.53% for RES recognition, which is suitable for the scenario of low-power requirements for RES recognition while guaranteeing high computing performance. This ensures both efficient resource utilization and optimal system performance for RES recognition.

The rest of this paper is organized as follows: Section 2 introduces RESs, the operational properties of CNNs and RNNs, and the 1D-CNN-LSTM algorithm constructed in this paper for RES identification. Section 3 introduces the design ideas and methods of the FPGA-based resource multiplexed computing acceleration platform. Section 4 presents the related experimental results. Section 5 is the discussion of the results, and Section 6 concludes this paper.

## 2. Related Works

### 2.1. Radar Emitter Signal Recognition

Early radar signals were single-carrier pulses without intra-pulse modulation, but with the advancement of radar technology, various modulations of radar signals began to be performed to improve the radar range of action and Doppler resolution. The five parameters (carrier frequency, amplitude, pulse width, direction of arrival, and signal time of arrival) of the conventional pulse description word (PDW) can no longer fully characterize the state information of the radar pulse. The RES intra-pulse modulation characteristics have consequently become an important parameter to describe the characteristics of the radar pulse. The main radar signal intra-pulse modulation methods are frequency modulation and phase modulation, which can be divided into continuous frequency modulation and discrete frequency modulation. Phase modulation mainly has two-phase coding, four-phase coding, and multi-phase coding [17].

Regardless of the modulation method, the modulation information is contained in the time domain signal or transform domain characteristics of the pulse. Traditional CNNs require uniformity in the dimensionality of the input time domain signal or transform domain features for RES identification. However, the pulse width, carrier frequency, amplitude, and signal bandwidth of the RESs are parameters that can change randomly; this requires that the algorithm model used for recognition must set redundancy according to the range of parameters required by the system and, thus, improve the applicability of the algorithm [18]. For example, the radar IF signal pulse width varies from a few microseconds to several tens of microseconds, and for some special functions of the radar signal, the signal pulse width may even reach the millisecond level. The carrier frequency ranges are generally in the 30 to 500 MHz range. Even without considering special requirements, if the signal is sampled for signal processing with a sampling rate that meets the bandwidth, the data length of the signal varies from several hundred to several tens of thousands of points. Whether the signal dimension is unified by using time domain or transform domain redundancy, it will inevitably result in wasted computational resources.

### 2.2. Convolutional Neural Networks

**Figure 2:**
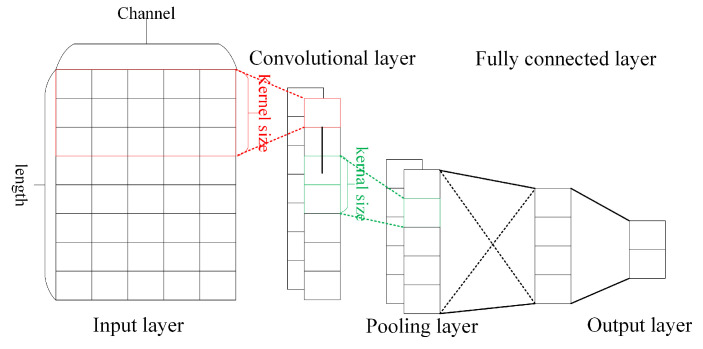
A typical 1D-CNN algorithm model.

A typical 1D-CNN algorithm model [19] is shown in Figure 2, which generally consists of an input layer, convolutional layer, pooling layer, fully connected layer, and output layer. In the actual algorithmic model, the convolutional and pooling layers are generally used alternately several times to form the depth structure.

Among them, the convolutional layer extracts the abstract features of the input data by convolutional operations, which are defined as follows:

(1) $$x_{j}^{l}=f\left(\sum_{i\in M}x_{i}^{l-1}⊗k_{ji}^{l}+b_{j}^{l}\right),$$

where $x_{j}^{l}$ denotes the channel output of the *j* channel of the *l* convolutional layer, f(•) denotes the activation function, *M* denotes the number of channels of the input data, $x_{i}^{l-1}$ denotes the *i* channel of the input data, ⊗ denotes the sequence convolution, $k_{ji}^{l}$ denotes the *i* channel of the *j* convolutional kernel of the *l* layer, and $b_{j}^{l}$ denotes the bias parameter of the *j* convolutional kernel of the *l* layer.

For the RES, if the quadrature dual-channel sampled IF data is used as the input of the convolution operation, the number of channels of the input data is fixed to 2, but the pulse length of the RES generally varies randomly. Therefore, using a fixed number of convolution kernels, the abstract features of the RES with randomly varying length and fixed number of output channels can be extracted by the convolution operation [20].

The main role of the pooling layer in a CNN is to obtain key features and achieve information dimensionality reduction. The pooling methods used (down-sampling methods) are generally average pooling and maximum pooling, where average pooling takes the average value of the features in the pooling window as the output, and maximum pooling takes the maximum value of the features in the pooling window as the output. The pooling operation is defined as follows:

(2) $${\hat{x}}_{j}^{l}=D\left(x_{j}^{l}\right),$$

where ${\hat{x}}_{j}^{l}$ denotes the pooled output of the *j* channel of the *l* layer, D(•) denotes the down-sampling function, and $x_{j}^{l}$ denotes the pooled input of the *j* channel of the *l* layer. From the definition of the pooling operation, it can be seen that the number of channels and the length of the output features of the pooling layer depend on the dimensionality of the input features. Therefore, the abstract features of the RES extracted by the convolutional layer are still abstract feature data with randomly varying lengths and a fixed number of channels after down-sampling by the pooling layer.

The fully connected layer is used to map the feature information extracted from the convolutional and pooling layers to a more separable space for the final classification output [21], and the fully connected operation is defined as follows:

(3) $$\begin{array}{cc} {\left(y_{1},y_{2},\ldots ,y_{m}\right)}^{T}= & W_{mj}{\left({\hat{x}}_{1}^{l},{\hat{x}}_{2}^{l},\ldots ,{\hat{x}}_{j}^{l}\right)}^{T}\\ & +{\left(b_{1},b_{2},\ldots ,b_{m}\right)}^{T}, \end{array}$$

where ${\left(y_{1},y_{2},\ldots ,y_{m}\right)}^{T}$ is the output of the fully connected operation, $W_{mj}$ is the weight matrix, $W_{mj}{\left({\hat{x}}_{1}^{l},{\hat{x}}_{2}^{l},\ldots ,{\hat{x}}_{j}^{l}\right)}^{T}$ is the expanded feature vector as the input to the fully connected operation, and ${\left(b_{1},b_{2},\ldots ,b_{m}\right)}^{T}$ is the bias vector. From the definition of the fully connected operation, it is clear that once the dimensionality of the weight matrix is determined, the dimensionality of the input features must be determined, which obviously cannot be adapted to the operation of data with random length radiation source signal features.

The above analysis leads to the following conclusion: the convolution and pooling operation layers of the 1D-CNN have no requirement on the length of the input signal, under the condition that the number of input data channels is determined. Since the convolutional computation uses weight sharing to extract data features, the change in the input data length does not affect the properties of the output features. Although the fully-connected layer can map the feature data to a separable space for classification and recognition, it cannot adapt to changes in the dimensionality of the input features. Therefore, when using a CNN to solve the problem of inconsistent input data dimensions, a redundancy mechanism must be used to unify the signal dimensions before they are fed into the algorithm model for processing. This will inevitably lead to a waste of computational resources. To solve this problem, we discuss the LSTM in the next section.

### 2.3. Long Short-Term Memory

**Figure 3:**
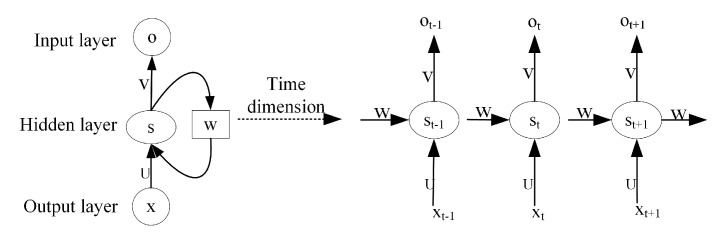
Recurrent neural network.

An RNN generally consists of an input layer, hidden layer and output layer [21], whose operations are expanded in the time dimension in Figure 3. RNN operations are defined as follows:

(4) $$\begin{array}{cc} s_{t} & =\left[W,U\right]{\left(s_{t-1},x_{t}\right)}^{T}, \end{array}$$

(5) $$\begin{array}{cc} o_{t} & =V(s_{t}), \end{array}$$

where *W* and *U* are the weight matrices of $s_{t-1}$ and $x_{t}$, respectively, and $V\left(•\right)$ is the activation function. The output $o_{t}$ of the RNN at time *t* depends on the state $s_{t}$ of the network at the current time, which is related not only to the current input $x_{t}$, but also to the network state $s_{t-1}$ at the time t−1 of the network. The network records the hidden state $s_{t}$ at time *t* and passes it to time t+1 until the last iteration, and the output of the network contains the state information of all historical moments, which enables the RNN to capture the order-dependent features such as location or time in the input data. Meanwhile, the cyclic structure of the RNN network itself determines its ability to adapt to random variations in the input sequence length.

(6a) $$\begin{array}{cc} f_{t} & =sigmoid(w_{f}\cdot \left[h_{t-1},x_{t}\right]+b_{f}); \end{array}$$

(6b) $$\begin{array}{cc} i_{t} & =sigmoid(w_{i}\cdot \left[h_{t-1},x_{t}\right]+b_{i}); \end{array}$$

(6c) $$\begin{array}{cc} o_{t} & =sigmoid(w_{o}\cdot \left[h_{t-1},x_{t}\right]+b_{o}); \end{array}$$

(6d) $$\begin{array}{cc} {\hat{c}}_{t} & =tanh(w_{c}\cdot \left[h_{t-1},x_{t}\right]+b_{c}); \end{array}$$

(6e) $$\begin{array}{cc} C_{t} & =f_{t}*C_{t-1}+i_{t}*{\hat{c}}_{t}; \end{array}$$

(6f) $$\begin{array}{cc} h_{t} & =o_{t}*C_{t} \end{array}$$

**Figure 4:**
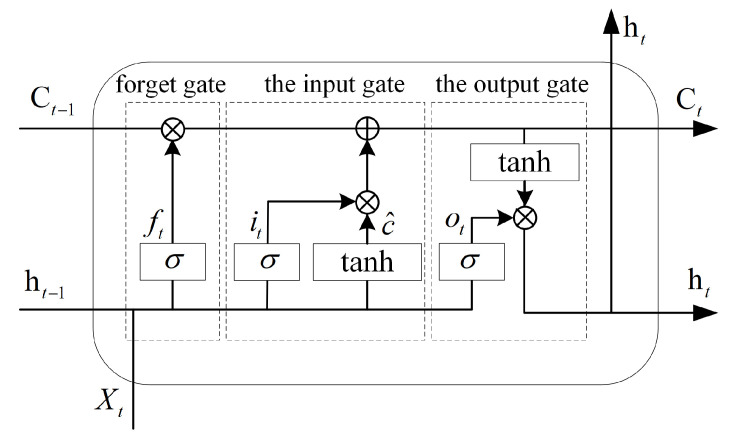
Structure of an LSTM neural network.

However, the simple RNN network structure often suffers from the problem of gradient dispersion and gradient explosion during the training process [22], which makes the network parameters fail to converge for a long time during the training process. The author proposed the LSTM [23], which solves this problem to a certain extent. The structure of the LSTM NN is shown in Figure 4, which introduces the concept of gate in the traditional RNN to control the opening of an information flow in the network cycle by simulating the characteristics of human memory to achieve the goal of local key information filtering and long-time-span feature synthesis. In (6), the relevant definitions of Figure 4 are explained. The three gates in the LSTM are the forget gate $f_{t}$, the input gate $i_{t}$, and the output gate $o_{t}$. All three gates are composed of sigmoid cells controlled by the current input $x_{t}$ and the previous time output $h_{t-1}$. The forget gate controls the opening of the historical state $C_{t-1}$ into the current state $C_{t}$, the input gate controls the opening of $x_{t}$ and $h_{t-1}$ into $C_{t}$, and the output gate controls the opening of $C_{t}$ into the current output $h_{t}$.

The LSTM not only increases the gating structure compared with the simple RNN, which improves the network trainability and sequence feature synthesis ability, but also inherits the characteristics of the simple RNN to adapt to the indefinitely long input. This makes the LSTM more suitable for synthesizing the information from the local radiation source signal features extracted by the convolution operation. The reason why the LSTM network is not used directly for signal feature extraction is that its structure determines that it can only be executed sequentially in the time dimension and is not suitable for parallel computing, and the parallelism of the algorithm is especially important for processing RESs with sequence lengths in the thousands [23].

### 2.4. 1D-CNN-LSTM

**Figure 5:**
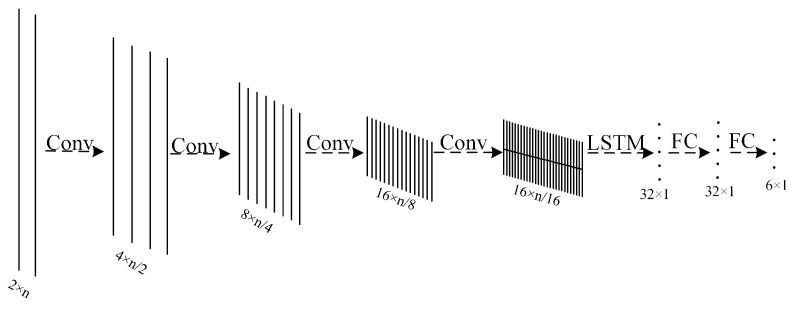
1D-CNN- LSTM neural network model.

We combined the characteristics of RESs with a large variation range of parameters, strong randomness, and 1D time domain sequence, using the good local feature extraction ability of CNNs. The LSTM is good at capturing time series information and can adapt to the random variation of the input data length and the fully connected network can map the features to the separable space. The 1D-CNN-LSTM model is constructed as shown in Figure 5; “n” represents the length of the RES with variable length. The network model directly processes the IF data of the RES, and completes the classification and identification of six different modulation types of RES, which include continuous wave (CW), binary frequency shift keying (BFSK) signals, binary phase-shift keying (BPSK) signals, quadrature phase-shift keying (QPSK) signals, linear frequency modulation (LFM) signals, and nonlinear frequency modulation (NLFM) signals [24].

The NN model consists of four convolutional layers, one LSTM layer, and two fully connected layers. The number of input channels of each convolutional layer is 2, 4, 8, and 16, the number of output channels is 4, 8, 16, and 32, and the length of the convolutional kernel is 15. Each convolutional layer uses a maximum pooling method with a pooling kernel length of 2 to reduce the dimensionality of the feature data, and the linear rectification function ReLU is used as the activation function. The input dimension of the LSTM layer is 32, and the size of the hidden layer of the LSTM is 32. The input of the first fully connected layer is the last updated hidden layer state vector of the LSTM, and the output dimension is 32×1. The input dimension of the second fully connected layer is the same as the output dimension of the first convolutional layer, and the output dimension is 6×1, which corresponds to six different modulation types of source signals. However, this process can be omitted when deploying the inference network on FPGAs, and the classification results can be derived from the numerical magnitude of the output of the fully-connected layer alone.

## 3. System Design and Structure

In this section, the design ideas and methods of the FPGA-based resource multiplexing computing acceleration platform will be presented, and the 1D-CNN-LSTM algorithm constructed in this paper for RES identification will be implemented, as shown in Figure 1.

### 3.1. One-Dimensional Discrete Convolution and Matrix–Vector Multiplication

**Figure 6:**
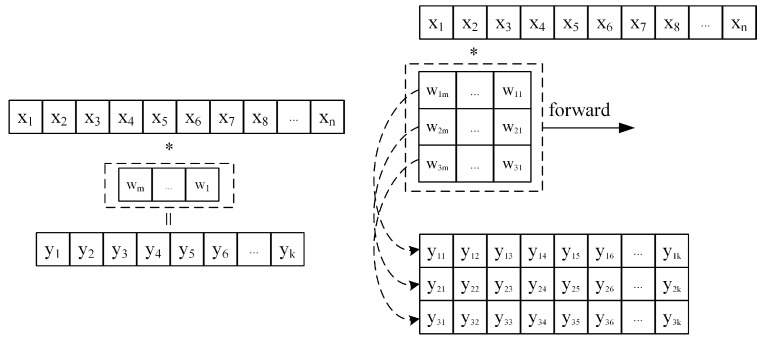
One-dimensional discrete convolution operation process. ∗ represents the inner product operation.

The 1D discrete convolution operation is an operation that computes the output feature sequence y(k) by sliding multiplication and accumulation of a fixed-length weight kernel w(m) with the input sequence x(n), as shown in Figure 6.

The calculation process shows that each result y(k) of the convolution calculation can be viewed as the inner product of vector $\left(w_{m},w_{m-1},\cdots ,w_{1}\right)$ and vector $\left(x_{k},x_{k+1},\cdots ,x_{k+m}\right)$. Similarly, when multiple convolution kernels are convolved with the input sequence at the same time, multiple output sequences can be obtained. For example, if the kernel size is 3, the operation process is shown in Figure 6. It can be seen that the result $\left(y_{1},y_{2},y_{3}\right)$ obtained from each sliding calculation of the convolution kernel is actually the result of multiplying the weight matrix and the vector $\left(x_{k},x_{k+1},\cdots ,x_{k+m}\right)$ formed by *w*.

**Figure 7:**
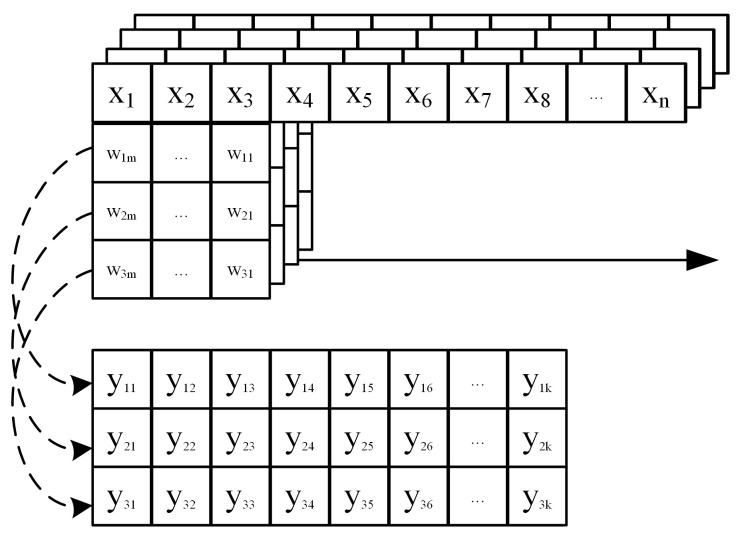
The actual operation process of 1D-CNN. Taking the 1D convolution operation with multi-channel input and multi-channel output with the number of input channels of 4 and a convolution kernel size of 3 as an example.

Through the above analysis, it is easy to find that the convolution operation of a multicore is actually composed of multiple matrix–vector multiplication operations. This makes it theoretically feasible to reuse FPGA hardware computing resources to achieve parallel acceleration of both convolutions in 1D-CNN and matrix–vector multiplications in LSTM [22]. However, the input data for the convolution operation in 1D-CNN is not a single channel, but the number of channels of input data increases as the number of convolution layers increases. Corresponding to the number of channels of input data, the dimension of convolution kernel increases, and the convolution result becomes the sum of the convolution results of each channel. The operation process in the actual 1D-CNN is shown in Figure 7.

### 3.2. Design of Systolic Array Structures

A systolic array [25] is a pipeline structure that can do multiple computations per memory access and consists of a set of interconnected elementary operators, each of which is capable of performing some simple operations. Because simple, regular communication and control structures have significant design and implementation advantages over complex ones, they can accelerate the execution of edge computing problems without increasing I/O requests [26]. First, 1D convolution and matrix vector multiplication operations are typically computationally constrained computations because the total number of computation operations is greater than the total number of input and output elements, and are well suited for parallel computation acceleration using systolic arrays. Secondly, because the structure of each processing element (PE) in the systolic array structure is relatively independent, it is less likely to cause wiring congestion when deployed on FPGAs, which helps to increase the operating frequency of the system.

**Figure 8:**
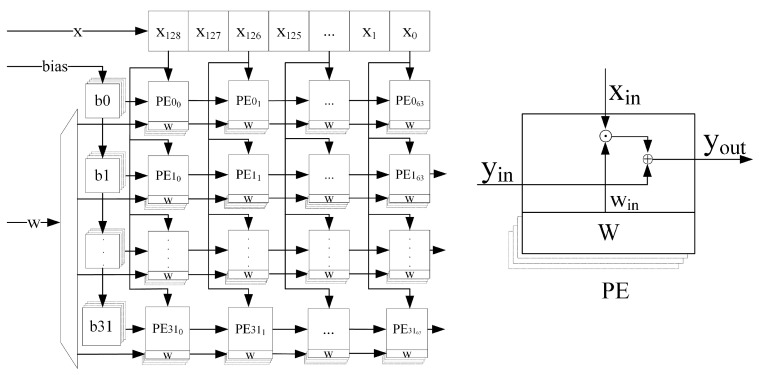
Systolic array structure and PE.

Based on the above advantages of systolic arrays, the systolic array structure shown in Figure 8 is designed to perform parallel acceleration of 1D convolutional and matrix vector multiplication operations in the CNN-LSTM. According to the 1D-CNN-LSTM structure constructed in this paper, the size of the systolic array is designed to be 32×64, which can support the parallel computation of 1D single-channel convolution with the length of the convolution kernel not larger than 64 and the number of kernels not larger than 32, or the parallel computation of matrix vector multiplication with the size of the weight matrix not larger than 32×64.

The structure of each PE of the systolic array is shown in Figure 8, including a bias input port $y_{in}$, a data input port $x_{in}$, a weight input port $w_{in}$, a weight cache unit, and a result output port $y_{out}$. Among them, the weight cache unit is composed of four registers with 16-bit width to store four different weight parameters, and the value in one of the registers is selected as a valid parameter to be used during the calculation as needed. In this paper, all the data involved in the operation are 16-bit fixed-point numbers, including 1-bit sign bits, 4-bit integer bits and 11-bit fractional bits. The basic operations performed by each PE in each clock cycle is:

(7) $$y_{out}=x_{in}*w_{in}+y_{in}$$

The weight matrices of the four gating coefficients are loaded into the weight cache unit at the same time during the iterative process of the LSTM loop, thus avoiding the system time delay caused by the update of the weight matrix at each loop and effectively improving the data throughput rate of the whole systolic array.

**Figure 9:**
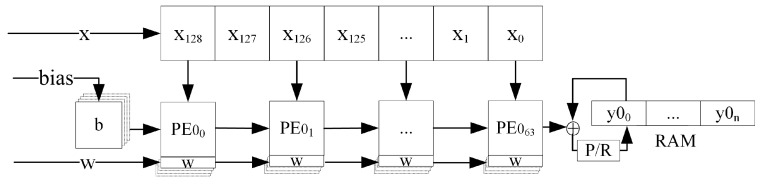
The cache structure of the systolic array computation results.

To compute multi-channel 1D convolution, the cache structure of the systolic array computation results is designed in this paper as shown in Figure 9.

When computing multi-channel convolution, the result of the previous channel is read from the corresponding address of the result cache RAM, added to the result of the current channel, and then stored back to the original address. The summation result can also be activated by the maximum pooling and linear rectification operations as needed before storing it back to the source address. The operation is defined as follows:

(8) $$\begin{array}{cc} \; & P/R(y_{k},y_{k+1},\cdots ,y_{k+l-1})\\ \; & =\left\{\begin{array}{cc} \max(y_{k},y_{k+1},\cdots ,y_{k+l-1}) & act_{en}=0\\ \max(y_{k},y_{k+1},\cdots ,y_{k+l-1},0) & act_{en}=1 \end{array}\right. \end{array}$$

where $y_{k}$ denotes the *k* pooling result, *l* denotes the pooling kernel size, and $act_{en}$ denotes whether to activate the result of the operation or not.

**Figure 10:**
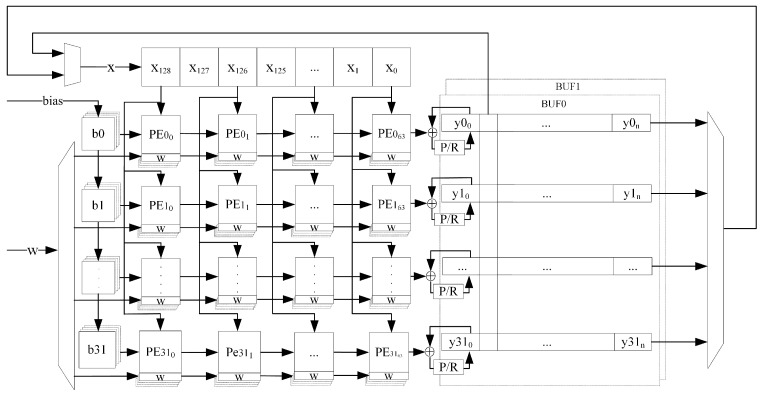
DMA double buffer.

For CNNs, the operation input of an intermediate layer is the operation result of the previous layer, and the operation output of that layer is the operation input of the next layer. Therefore, a single structure of systolic result cache is not enough to support multi-layer CNN operations. To solve this problem, we design a direct memory access (DMA) [27] double buffer to achieve the systolic operation result reuse seen in Figure 10.

The result cache array consists of two sets of 32 channels of RAM with the same structure, BUF0 and BUF1. When one of the BUF is used as the input source for the systolic operation, the other is used as the result cache. Of course, both of them support both single-channel row outputs and multi-channel column outputs, where the multi-channel column output function is used to realize the last convolutional layer operation result as the input of LSTM and the reuse of matrix vector multiplication results.

### 3.3. LSTM Neural Network State Update

**Figure 11:**
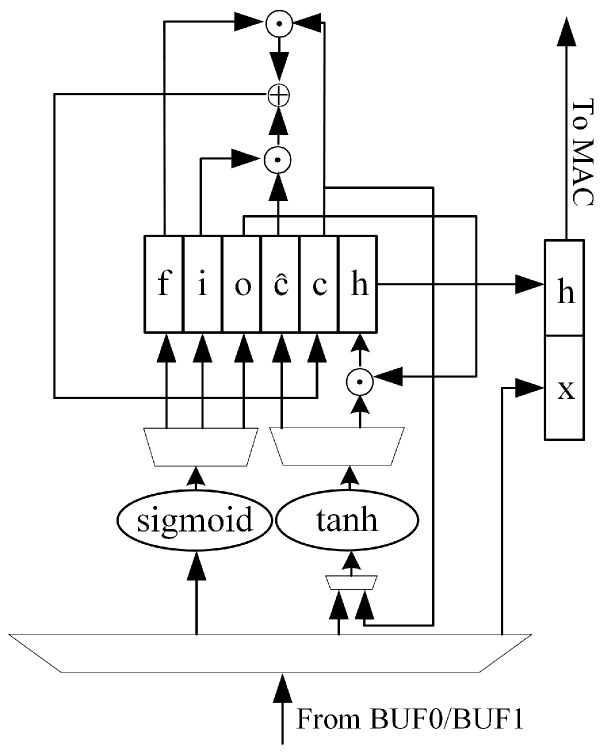
The state update of LSTM neural network.

To cooperate with the systolic array to complete the whole LSTM operation process, we design the operation module shown in Figure 11 to complete the state update, according to the LSTM operation definition.

The module reads the CNN result $x_{t}$ from the result cache of the pulsating array, and then loads it into the pulsating array for matrix–vector multiplication after forming the $\left[h_{t-1},x_{t}\right]$ vector with the LSTM output $h_{t-1}$ at the previous time. Then, it reads the result from the result cache of the pulsating array and sends it to the sigmoid or tanh activation unit.

**Figure 12:**
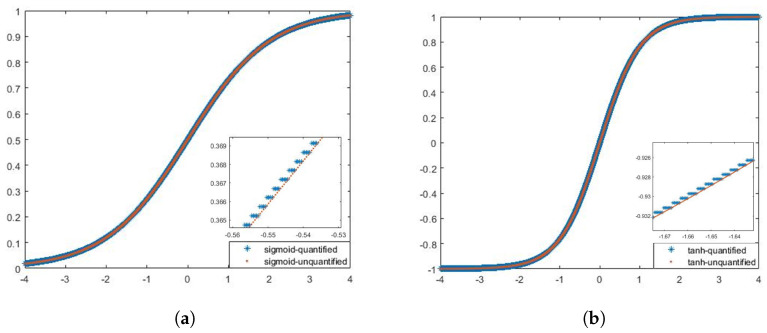
Quantization process of two nonlinear activation units. (**a**) Sigmoid nonlinear activation units. (**b**) Tanh nonlinear activation units.

The two nonlinear activation units, sigmoid and tanh, are implemented using a table lookup. The values of the two functions are quantized and stored in the ROM in advance, and when the nonlinear activation of the matrix vector multiplication results is required, they are first converted to the corresponding ROM addresses and then read directly from the ROM to obtain the function values. We use 16-bit fixed-points to quantize the nonlinear functions. The quantization process intercepts the part of the independent variables of sigmoid and tanh in [−4,4) for sampling, and the part of the independent variables beyond the sampling range is taken as the boundary point in Figure 12.

### 3.4. Acceleration of the Overall Architecture of the Platform

**Figure 13:**
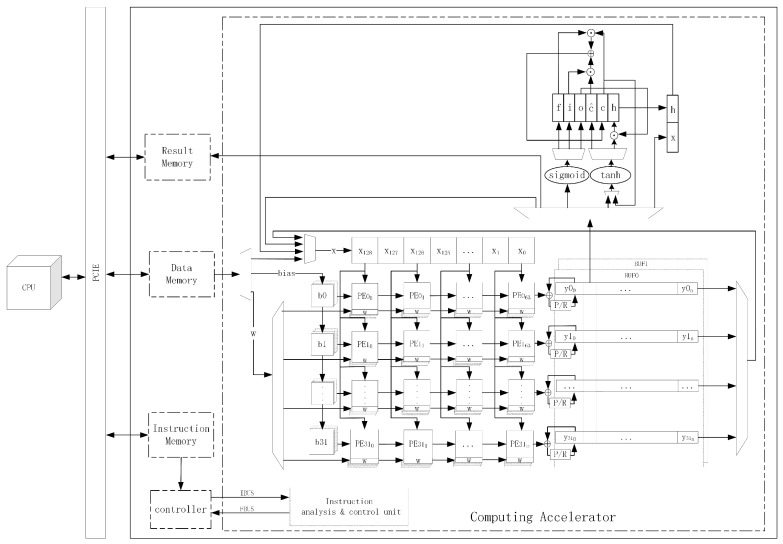
1D-CNN-LSTM resource multiplexing computational accelerator framework.

Combined with the designed systolic array and the LSTM state update circuit, we designed a 1D-CNN-LSTM resource multiplexing computational accelerator using a heterogeneous computational architecture of a Von Neumann-like system [28], as shown in Figure 13.

The accelerator consists of two parts: a general-purpose CPU and an FPGA, which realize data interaction through a peripheral component interconnect express (PCIE) bus. The general-purpose CPU is used to analyze the NN structure, generate corresponding operation instructions, and load them into the instruction memory on the FPGA side. Then, the RES data to be identified is loaded into the FPGA data memory, and the identification results are read out from the memory after the operation is completed. The FPGA includes five modules: instruction memory, data memory, result memory, controller, and operator. The controller module reads the operation instructions from the instruction memory, which, in turn, controls the operator module to complete the corresponding calculation, and writes the final operation results to the result memory for the CPU to read.

For the computational acceleration platform designed in this paper, a dedicated instruction set with a 64-bit bit width was developed to support 1D convolution, matrix multiplication, LSTM operations, and data read/write functions, as shown in Table 1.

The instruction content is divided into four parts: instruction ID, function code, operand length, and data source start address. The instruction ID field, as a unique code for each instruction, is used to distinguish different operations. The function code field is used to control some optional functions during the instruction execution. The operand length field is used to specify the total amount of data for an instruction operation. The source address field is used to specify the starting address of the operand. The entire instruction set is divided into four types: systolic array parameter loading instruction, systolic array data loading instruction, LSTM operation instruction, and data moving instruction. Among them, the systolic array parameter loading instructions include two instructions: weight parameter loading and bias parameter loading. The systolic array data load instructions include five instructions: loading from data memory, loading from BUF0 row, loading from BUF1 row, loading from BUF0/BUF1 column, and loading from LSTM output. With LSTM operation instructions and data moving instructions, the entire acceleration platform-specific instruction set consists of nine instructions.

When the hyperparameters of the NN model, such as the number of convolutional layers and size of the convolutional kernel, or the model parameters need to be adjusted, the computational acceleration platform designed in this paper does not need to redesign the accelerator hardware logic. It only needs to make adjustments to the instruction content according to complete the rapid deployment of the new algorithm model, greatly improving the radar emitter source identification system. This is particularly important for deep learning algorithms to adapt to the rapidly changing electromagnetic environment.

## 4. Experiments

In this section, the 1D-CNN-LSTM model and its experiments based on FPGA deployment are presented. The details of the experiments are explained, the experimental results and performance analysis are shown, and a comparison of different works is performed to evaluate the performance of our approach.

In the experiment, a computer with Intel i7-10700@2.9GHz CPU and the PyTorch deep learning framework was used. We used Vivado 2018.3 development tool to implement and deploy the algorithm on Xilinx XCKU040-ffva1156-2-i for the 1D-CNN-LSTM acceleration designed in this paper. The entire FPGA accelerator system computing unit frequency was 120 MHz. In the comparative experiment, NVIDIA GPU RTX 3090 was used.

### 4.1. Dataset

To demonstrate the effectiveness of our designed RES identification system, RESs were are generated according to Table 2. We randomly generated six modulation types by simulation, with 3000 sample signals for each type of RES. We divided the 18,000 signals into two parts—the training set and the test set—according to the ratio of 5:1, for the training and generalization performance testing of constructing a 1D-CNN-LSTM model.

### 4.2. Result

**Figure 14:**
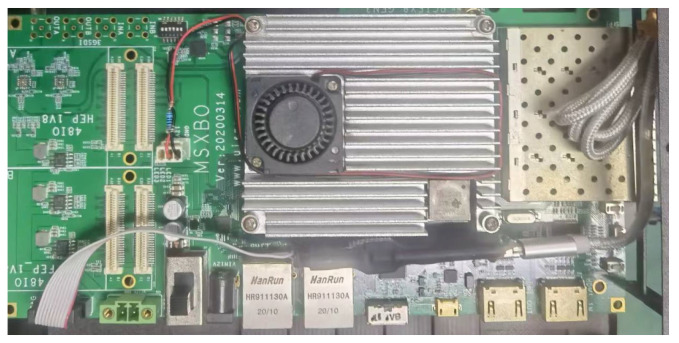
FPGA Experiment platform, connected to CPU by PCIE bus.

**Figure 15:**
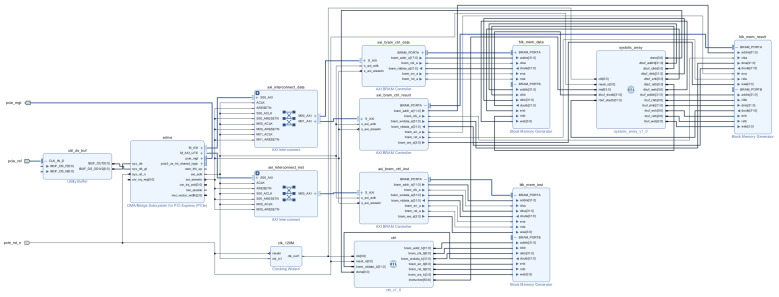
FPGA hardware design schematic.

The physical object of the designed FPGA acceleration for RES identification is shown in Figure 14. In the Vivado 2018.3 development environment, the detailed FPGA hardware design part of the acceleration is shown in Figure 15.

**Figure 16:**
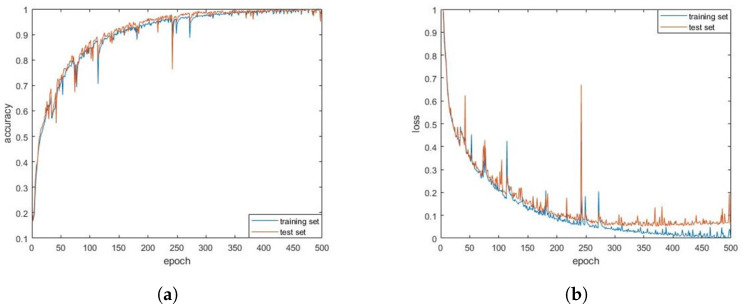
(**a**) Normalized recognition accuracy and loss values for training sets; (**b**) normalized recognition accuracy and loss values for testing sets.

**Figure 17:**
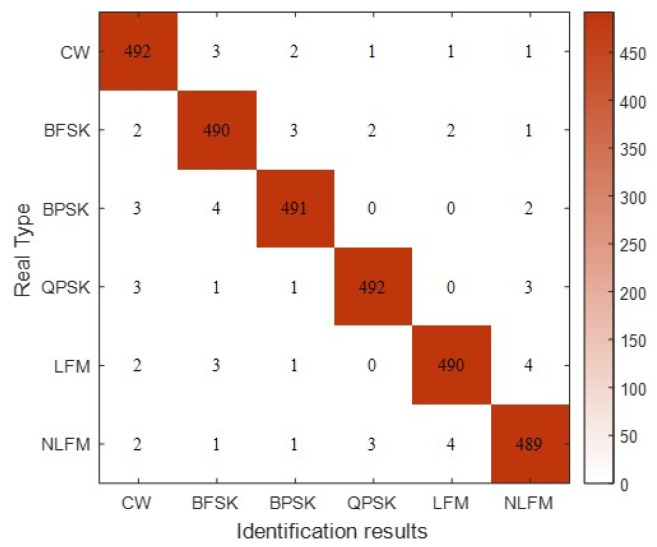
Confusion matrix for each type of recognition result.

We used the the Pytorch deep learning framework to train the constructed 1D-CNN-LSTM model. The normalized recognition accuracy and loss values of the algorithm model for the training and test sets during 500 iterations of the training set are shown in Figure 16. At the end of the training, the recognition accuracy of the model for the training and test sets reached 100% and 97.27%, respectively. To demonstrate the identification of the various categories, we present the confusion matrix in Figure 17. We found that between LFM and NLFM, BPSK and QPSK were more difficult to identify. Other types were better identified, and the identification results were relatively evenly distributed.

**Figure 18:**
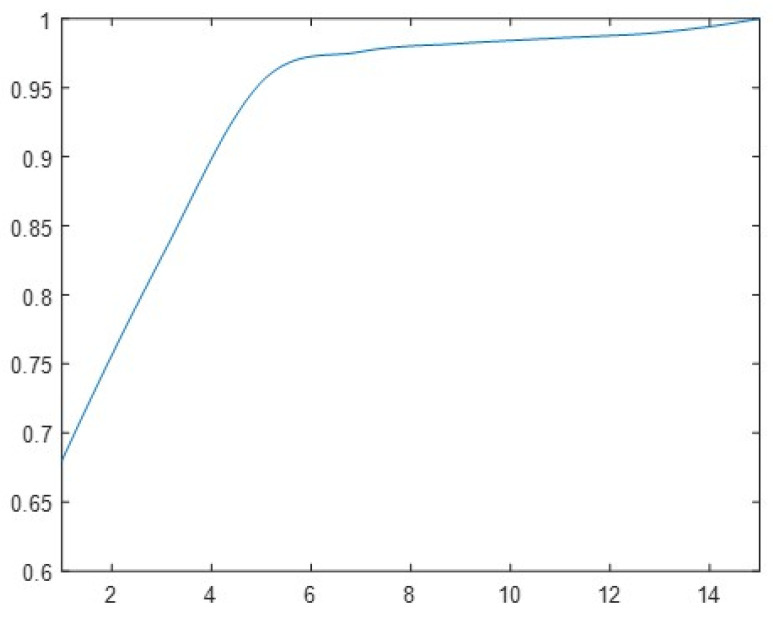
Recognition results with different SNR.

The above experiments were all completed under the random SNR of 5–15 dB for recognition. To identify the RESs for different SNR, the SNR of the test set data samples was reset, and the recognition experiments were conducted between 1–15 dB, and the results are shown in Figure 18.

It can be seen that the recognition rate of the algorithm exceeded 80% when the SNR was higher than 5 dB, recovered to the level after the end of training, and the lowest still had an 82.7% accuracy in the case of a low SNR of 3 dB. This indicates that the 1D-CNN-LSTM network has better adaptability in completing the RES recognition under different SNRs.

The trained algorithm model was coded and the parameters quantized, and then deployed on the computational acceleration platform designed in this paper for testing. For the training and test sets constructed in this paper, the processing performance of a total of 18,000 signals was tested with the Pytorch deep learning framework on the CPU and the FPGA computing acceleration platform designed in this paper as acceleration devices to run the same models. The comparison of recognition accuracy is shown in Table 3.

The experimental results show that the recognition rate of the NN deployed on the FPGA computing platform for the training and test set signals was reduced by 0.46% and 0.74%, respectively, compared to that under the Pytorch deep learning framework. The recognition rate decreased due to the quantization error of both parameters and signals. When implemented on FPGAs, we quantized to 16 bits to reduce the computation and design complexity for hardware implementation. Moreover, the acceleration platform designed in this paper needs to adapt to the inference computation of different structural NN models. Hence, the quantization of NN models was performed with 16-bit fixed-point arithmetic considering the quantization accuracy and model adaptation capability. For our 1D-CNN-LSTM model, the recognition accuracy is considerably high, indicating that the algorithm can be implemented on the FPGA accelerated platform to perform RES recognition.

## 5. Discussion

### 5.1. Adaptability for Varying Pulse Width

To demonstrate the adaptability of the 1D-CNN-LSTM model in processing RESs with randomly varying pulse widths, we uses nine sets of RES samples with the same range of other parameters as the training set and pulse widths distributed between 1 μs and 10 μs for recognition accuracy testing.

**Figure 19:**
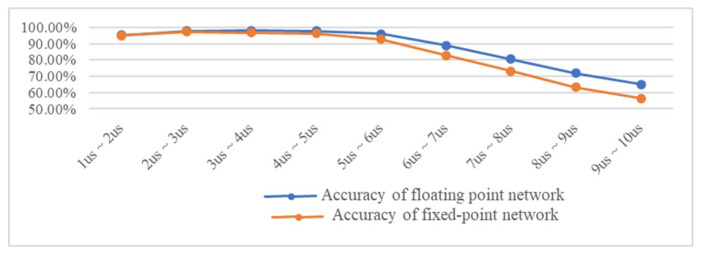
Recognition accuracy for different pulse widths.

The test results in Figure 19 show that, regardless of the inference environment, the recognition accuracy of the model started to show a decreasing trend after the signal pulse width exceeded the distribution of the training set parameters. However, the recognition rate of the model remained at a high level when the pulse width reached twice the distribution of the training set. Due to the training set with pulses of 1–5 μs RES, it inevitably led to a decrease in recognition rate when the training set range was exceeded. However, recognition could still be performed with increasing variation in pulse length, and even 9–10 μs could reach more than 55%. It is worth noting that the CNN-LSTM has the advantage of being adaptable to pulse widths beyond the training set distribution, which is not achieved by a single CNN.

### 5.2. Efficiency Analysis of Acceleration

To evaluate FPGA acceleration performance, the processing performance was tested on the CPU, GPU, and the FPGA computing acceleration platform designed in this paper. On the test set, we ran the same model separately to process the same calculation for inference.

As shown in the test results of Table 4, the processing speed and data throughput of the FPGA acceleration platform were 4.16 times higher than that of the CPU. When in the GPU environment, the Pytorch deep learning framework could take full advantage of the GPU’s multi-core parallel processing, which greatly improved the system’s data throughput rate to 16.73 GOPS, which was about 2.3 times higher than that of the FPGA compute acceleration platform, but the system power consumption also increased. In terms of energy efficiency, the FPGA computing acceleration platform designed in this paper improved 73 times and 9.125 times compared to the CPU and GPU, respectively. Our FPGA implementation showed much higher energy efficiency when still meeting the data processing requirement.

### 5.3. Resource Utilization

To represent the advantages of the acceleration performance proposed in this paper, the following resource utilization analysis was performed after using measures such as fixed-point and systolic array structures. The FPGA hardware resource consumption is shown in Table 5.

The FPGA chip used in this paper has a total of 1920 DSP hardware computing resources, and the systolic array was used in the acceleration optimization with a scale of 32×64. If calculated according to the consumption of one DSP resource per PE, the entire on-chip resources are far from sufficient, but other logic resources can be fully used. For example, through Vivado, we obtained LUT usage of 71%, FF usage of 39%, BRAM usage of 79%, and DPS usage of 100%. These resources can also be integrated to complete the multiplication and addition operations to satisfy the requirements. It also demonstrates one of the major advantages of FPGAs in the design process of large-scale integrated circuits.

### 5.4. Comparison with Other FPGA Implementations

The proposed implementation in this paper was compared with some existing FPGAs and the results are shown in Table 6.

For FPGA designs, resource consumption varies across architectures. The excellent throughput performance achieved in hardware may be mainly due to the heavy use of hardware resources. Compared to other work, our acceleration in inference phase achieved 95.5% of RES recognition and had a more reasonable relationship between power consumption, resource consumption, and throughput, thus enabling real-time processing.

## 6. Conclusions

In this paper, we constructed a 1D-CNN-LSTM model, which can be deployed on an FPGA-based resource multiplexing computing acceleration platform while ensuring high RES recognition accuracy. The experimental results show that the model is highly adaptable for the recognition of RES pulses with randomly varying lengths. For the computational acceleration platform, we designed the pulsed array to realize the parallel acceleration of both 1D convolution and vector matrix multiplication operations, which reduced the processing latency of the RES identification system and improved the utilization of FPGA computational resources at the same time. Further experiments and correlation analysis illustrate the contribution of each of our improvements and demonstrate their effectiveness. Deploying the system on a Xilinx XCKU040 FPGA development board achieved a data throughput rate of up to 7.34 GOPS with a power consumption of 5.022 W at a recognition rate of 97.53% for the RES modulation method. In conclusion, our experimental results demonstrate that the FPGA-based CNN-LSTM RES recognition system effectively meets the requirements of low-power RES scenarios while maintaining computational accuracy. With these features, our solution holds significant potential for RES recognition.

In the future, we will continue to improve our models in terms of recognition algorithms to improve the accuracy of RES recognition. Additionally, we will keep optimizing our FPGA-based hardware acceleration platform for more NN models and accelerated computation for low-power implementation.

## Figures and Tables

**Table 1:** Instruction examples from the hardware instruction set.

Instruction ID	Function Code	Operand Length	Source Address
63:60	59:36	35:18	17:01 ^1^

^1^ Numbers indicate the start and end positions of the instructions.

**Table 2:** Specific parameters of the radar emitter signals.

Type	CW	BFSK	BPSK	QPSK	LFM	NLFM
Carrier Frequency	100–400 MHz					
Pulse Width	1–5 μs					
Band Width	–	5–50 MHz	–	–	5–50 MHz	5–50 MHz
Code	–	13-digit Barker code	13-digit Barker code	16-digit frank code	–	–
Frequency of Sample	500 MHz					
Signal to Noise Ratio	5–15 dB					

**Table 3:** Comparison of recognition accuracy in different environments.

Inference Environment	Training Set	Testing Set
Pytorch deep learning framework	100%	97.27%
FPGA acceleration platform	99.54%	96.53%

**Table 4:** CPU, GPU, and FPGA computing acceleration platform processing performance.

Platform	Calculation	Processing Time (s)	Throughput (GOPS)	Consumption (W)	Energy Efficiency (GOPS/W)
CPU	108.28 G	61.36	1.76	81.19	0.02
GPU	108.28 G	6.47	16.73	102.5	0.16
FPGA	108.28 G	14.75	7.34	5.022	1.46

**Table 5:** FPGA resource utilization.

Logic	LUT	FF	BRAM	DSP
Results	171,497 (71%)	188,405 (39%)	472.0 (79%)	1920 (100%)

**Table 6:** Performance comparison of our work with other accelerators.

	Our Work	[10]	[11]	[29]	[30]
Platform	Xilinx XCKU040	Xilinx AC701	Xilinx ZCU102	Xilinx ZCU102	Artix7 TSBG484
Frequency (MHz)	120	200	153.9	300	100
Throughput (GOPS)	7.34	23.06	-	102	22
Power (W)	5.022	3.407	4.7	11.8	7.53
Energy efficiency (GOPS/W)	1.46	6.77	3.49	8.64	2.92

## Data Availability

Not applicable.
